# Proactive Deployment of Aerial Drones for Coverage over Very Uneven Terrains: A Version of the 3D Art Gallery Problem

**DOI:** 10.3390/s19061438

**Published:** 2019-03-23

**Authors:** Andrey V. Savkin, Hailong Huang

**Affiliations:** School of Electrical Engineering and Telecommunications, University of New South Wales, Sydney 2052, Australia; a.savkin@unsw.edu.au

**Keywords:** aerial drones, UAVs, art gallery problem, drone art gallery theorem, aerial surveillance, proactive deployment, coverage, cellular networks, combinatorial geometry, computational geometry

## Abstract

The paper focuses on surveillance and monitoring using aerial drones. The aim is to estimate the minimal number of drones necessary to monitor a given area of a very uneven terrain. The proposed problem may be viewed as a drone version of the 3D Art Gallery Problem. A computationally simple algorithm to calculate an upper estimate of the minimal number of drones together with their locations is developed. Computer simulations are conducted to demonstrate the effectiveness of the proposed method.

## 1. Introduction

Aerial Drones or Unmanned Aerial Vehicles (UAVs), which were initially used in military applications, have now been applied widely in various civilian tasks [1], such as infrastructure inspection [2], wireless communication support [3], parcel delivery [4,5,6,7], traffic monitoring [8], surveillance [9], and 3D surface reconstruction of large-scale elements which is of interest in different fields including topography, archaeological research and reconstruction of urban textures or industrial installations [10,11,12,13]. An important class of problems is the deployment of aerial drones for coverage of a certain ground area. In some such problems, drones are deployed to provide communications to users in either disaster areas or during occasional events attracting a lot of participants [3,14,15,16,17,18]. In other problems, the aim is to ensure monitoring of the ground area [19,20,21,22,23,24].

In surveillance and monitoring applications, aerial drones are equipped with some specific sensors, such as video cameras. They fly into the sky and monitor ground targets of interest, such as humans, buildings, pipelines, roads, vehicles, landmarks, and animals, for the purpose of surveillance, security, etc. [20,21,22,25]. A common scenario is when each drone is equipped with a ground facing video camera with some visibility angle. The video camera can see a circular area on the ground and the radius of this area depends on the altitude and the visibility angle. If a point is within this circle, it is considered to be covered by the drone. The altitude of each drone must be in a given range. A typical problem is to deploy the minimal number of drones necessary to cover a given ground area. A well-studied scenario is using a fleet of drones to monitor a given ground area and a general approach is to partition the area first and then plan paths for drones in the sub-areas [26]. A shortcoming of this partition-based method is that it does not guarantee the coverage of any point of the area at any time. Once the motion pattern of the drones is learned, intruders may be able to avoid the monitoring. Thus, a stronger requirement of monitoring applications is to ensure that any point of the area is seen from some drone and a very difficult and practically important extension of this problem is deployment of aerial drones over very uneven terrains, rather than ground areas. Uneven terrains are geometrically complex environments that cannot be approximated with a sufficient accuracy by a plane. In such environments, the visibility cones of drones’ video cameras may be occluded by buildings, walls, mountains, hills, etc. Very uneven terrains are quite typical for dense urban areas with tall buildings and narrow streets [18,27]. This problem will get especially important in the future with the use of small low flying drones [27]. One approach to this problem is to first find a set of positions such that every point on the terrain can be seen from at least one of the positions in the set [28,29] and then construct a tour for the drone to visit these positions such that every position is visited exactly once and the tour completion time is minimized. In contrast to [28,29], this paper focuses on the scenario where any point on the terrain must be monitored all the time. To achieve this, a constructive solution of this UAV deployment problem that may be called a drone version of the three-dimensional (3D) Art Gallery Problem is presented. The Art Gallery Problem is a well-known problem of combinatorial/computational geometry that deals with determining the minimal number of observers necessary to cover an art gallery room such that every point is seen by at least one observer. This problem was formulated by Victor Klee in 1973 [30] and is well studied, especially for the two-dimensional case [30,31,32]. In [33], the Art Gallery Theorem was published that gives an upper bound on the minimal number of observers in the 2D Art Gallery Problem. The proof of [33] was later simplified in [34] via a so-called 3-coloring method. In [35], a 3D version of the Art Gallery Problem was considered where observers are to be placed on uneven terrain modelled as a non-convex polytope. In this paper, a drone version of the 3D Art Gallery Problem in which observers (aerial drones) are to be placed above uneven terrain in a certain range of altitudes (not on the terrain as in [35]) is considered. Moreover, unlike [35], visibility cone constraints should be satisfied. An upper estimate of the minimal number of drones necessary together with an algorithm of calculating drone locations is given. This approach uses 3-coloring method of [34].

The remainder of the paper is organized as follows. The model of very uneven terrains and the problem statement are presented in Section 2. The proposed deployment algorithm together with the main result are given in Section 3. Section 4 presents simulations of the developed drone deployment algorithm. Finally, Section 5 provides a brief conclusion together with some possible directions of future research.

## 2. Problem Statement

Let (x,y) be Cartesian coordinates on the ground plane and *z* be the coordinate axis perpendicular to the ground plane. A terrain is a graph of a function F(x,y) that assigns to every point (x,y) on the ground plane an elevation z=F(x,y). The case F(x,y)=0 for all (x,y) corresponds to a perfectly flat (even) terrain. Moreover, let D be a given bounded subset of the ground plane z=0. The objective is to deploy several aerial drones to monitor the corresponding area of the terrain that is the set $\hat{D}:=\left\{(x,y,F(x,y))\right\}$ where (x,y)∈D. Also, let $Z_{min}$ and $Z_{max}$ be given minimum and maximum altitudes for drone deployment, $Z_{max}>Z_{min}>0$. Assume that each drone can be deployed only at some points $\left(x_{d},y_{d},z_{d}\right)$ such that

(1) $$\left(x_{d},y_{d}\right)\in D,\;\;\;\;z_{d}\in \left[Z_{min},Z_{max}\right].$$

Furthermore, let $\rho_{ij}$ be the distance between the drones *i* and *j*, and $\rho_{i}$ be the minimum distance between the drone *i* and the terrain. The following safety constraints should hold:

(2) $$\rho_{ij}\geq c_{1},\;\;\;\;\rho_{i}\geq c_{2}$$

where $c_{1}>0$ and $c_{2}>0$ are some given safety margins. The requirements (2) allow the avoidance of collisions of drones with the terrain and each other, which is very important in navigation and deployment, see e.g., [36,37,38,39].

**Figure 1:**
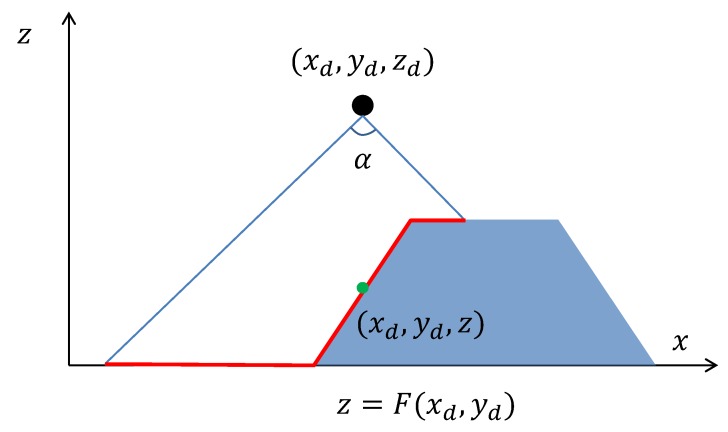
The region a drone can see.

Moreover, drones have a given observation angle 0<α<π, which defines the visibility cone of each drone, so that a drone with the coordinates $\left(x_{d},y_{d},z_{d}\right)$ can only see points (x,y,z) of the terrain that are inside of the circle of radius

(3) $$\begin{array}{c} r\left(z\right):=\tan\left(\frac{\alpha }{2}\right)\left(z_{d}-z\right) \end{array}$$

centered at $\left(x_{d},y_{d},z\right)$ where $z<z_{d}$. A point *P* on the terrain is visible from a drone located at the point *D* if *P* is inside of the visibility cone of the drone, and there is not any other point of the terrain on the straight line segment (D,P), see Figure 1.

**Definition** **1.** *Deployment of several drones is said to be covering if the constraints (1), (2) are satisfied and every point on the terrain region $\hat{D}$ is visible to at least one of the drones.*

**Figure 2:**
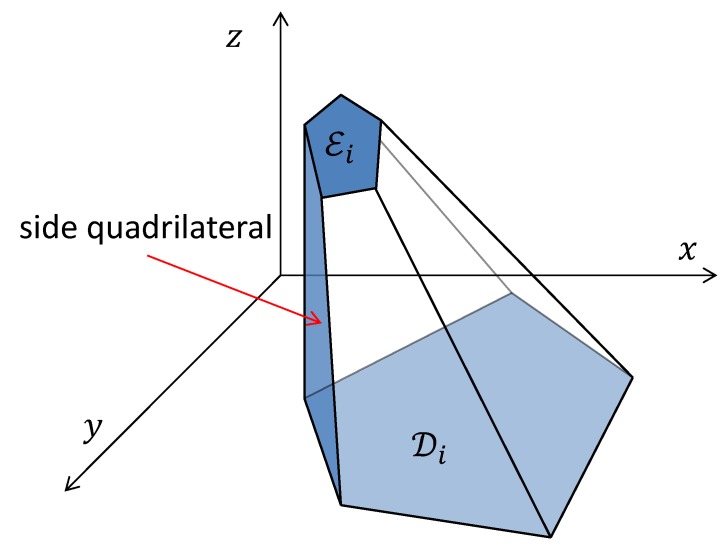
An illustration of a very uneven area.

**Terrain Model:** This paper considers the following class of very uneven terrains. First it is assumed that the set D is a polygon with *n* vertices. Furthermore, let $D_{1},\ldots ,D_{k}$ be some non-overlapping polygons inside D with $n_{1},\ldots ,n_{k}$ vertices, respectively. These polygons represent very uneven areas inside D such as buildings, hills, mountains, walls, etc. These areas are modelled as polytopes as follows. Let $E_{1},\ldots ,E_{k}$ be some other polygons inside the polygons $D_{1},\ldots ,D_{k}$, respectively. It is assumed that each polygon $E_{i}$ has the same number $n_{i}$ of vertices with the polygon $D_{i}$. It should be pointed out that $D,D_{1},\ldots ,D_{k}$ and $E_{1},\ldots ,E_{k}$ may be non-convex. Each $D_{i}$ represents the “base” face of the polytope modelling the corresponding very uneven area whereas $E_{i}$ represents its “top” face. Furthermore, it is assumed that this polytope has $k_{i}$ “side” faces. Each “side” face is a convex quadrilateral with one side that is a side of $D_{i}$ and with the opposite side that is a side of $E_{i}$, see Figure 2. These quadrilaterals are called side quadrilaterals. Notice that the case when some side of $E_{i}$ is a subinterval of $D_{i}$ means that the corresponding side face is a vertical wall. Also, the planes of the “top” faces are not assumed to be parallel to the ground plane z=0, see Figure 2. However, they are assumed to be not orthogonal to it. For example, a standard rectangular building is modelled by two identical rectangles $E_{i}$ and $D_{i}$. Moreover, it is assumed that the rest of polygon D that is outside of the very uneven areas $D_{1},\ldots ,D_{k}$, is "relatively even". More precisely, suppose that the following assumption holds.

**Assumption** **1.** *Any point (x,y,z) of the terrain outside the "very uneven" areas satisfies*

(4) $$\begin{array}{c} |z|\leq \epsilon , \end{array}$$

*where ϵ>0 is some given constant. Moreover, any two such points outside of the very uneven areas of the terrain satisfy the following constraint: the angle between the straight line connecting these two pints and the ground plane is less than $\beta :=(\frac{\pi }{2}-\frac{\alpha }{2})$.*

It is also assumed need the following technical assumptions that usually hold in practice.

**Assumption** **2.** *The inequality $\epsilon +c_{2}\leq Z_{min}$ holds.*

**Assumption** **3.** *The altitude of any point (x,y,z) corresponding to any vertex of any $E_{i}$ satisfies z>ϵ.*

The problem studied in this paper that may be called **the drone version of the 3D Art Gallery Problem** can be stated as follows.

**Problem Statement:** What is the minimum number of aerial drones for which covering deployment exists and where should they be deployed? Moreover, it is preferred not only to deploy the minimum number of drones but also deploy the drones at as low altitudes as possible to make them closer to the observed region of the terrain.

## 3. Deployment Algorithm

The deployment algorithm requires some geometric constructions. Let P be the polygon that is obtained from the polygon D with all the interior points of the polygons $D_{1},\ldots ,D_{k}$ taken away. It is a non-convex polygon with *k* “holes” and $n+n_{1}+\cdots +n_{k}$ vertices. The proposed algorithm consists of the following steps.

**Figure 3:**
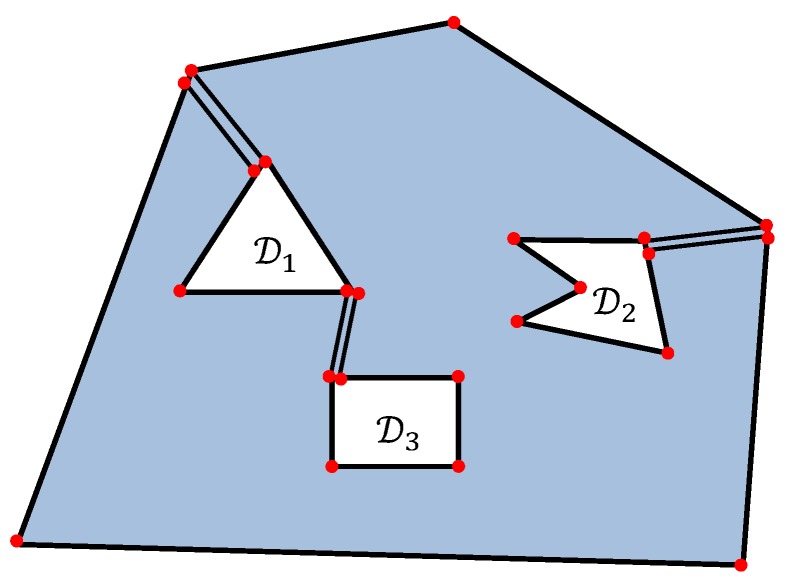
The construction of polygon Q by adding *k* non-intersecting diagonals to the polygon P.

**Step A1:** Choose *k* non-intersecting diagonals of the polygon P that cut it into a polygon Q without “holes”. More precisely, each of the *k* diagonals connects either two vertices of $D_{i}$ and $D_{j}$ for some i≠j or two vertices of $D_{i}$ and D. Now consider each of the *k* diagonals as two different sides of the polygon Q and each vertex of this diagonal as two different vertices of the polygon Q, see Figure 3. Hence the polygon Q is a polygon without “holes” with $n+n_{1}+\cdots +n_{k}+2k$ vertices.

**Figure 4:**
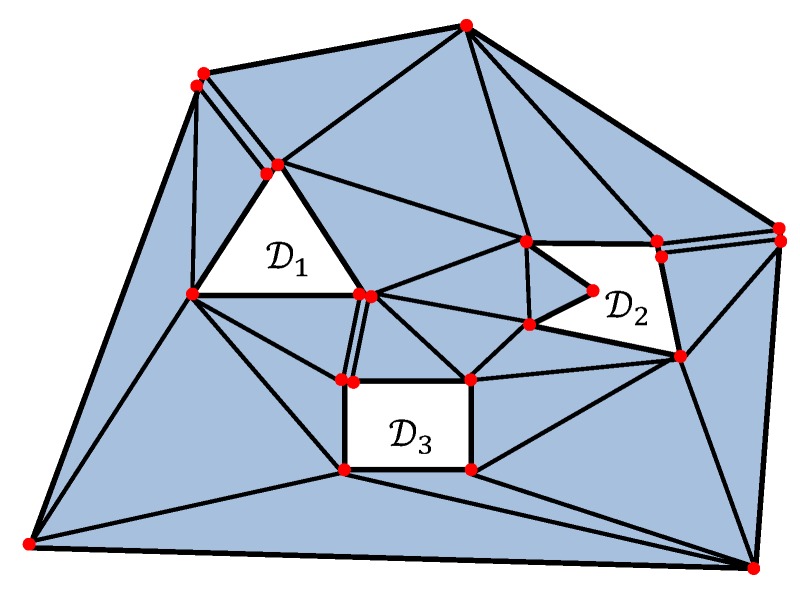
The triangulation T of the polygon Q.

**Step A2:** Make some triangulation T of the polygon Q. It means that Q is cut into triangles of vertices of which are vertices of the polygon Q, and all sides are either sides of Q or its non-intersecting diagonals, see Figure 4. Since Q has $n+n_{1}+\cdots +n_{k}+2k$ vertices and no “holes”, the number of triangles in any such a triangulation T is $n+n_{1}+\cdots +n_{k}+2k-2$.

**Figure 5:**
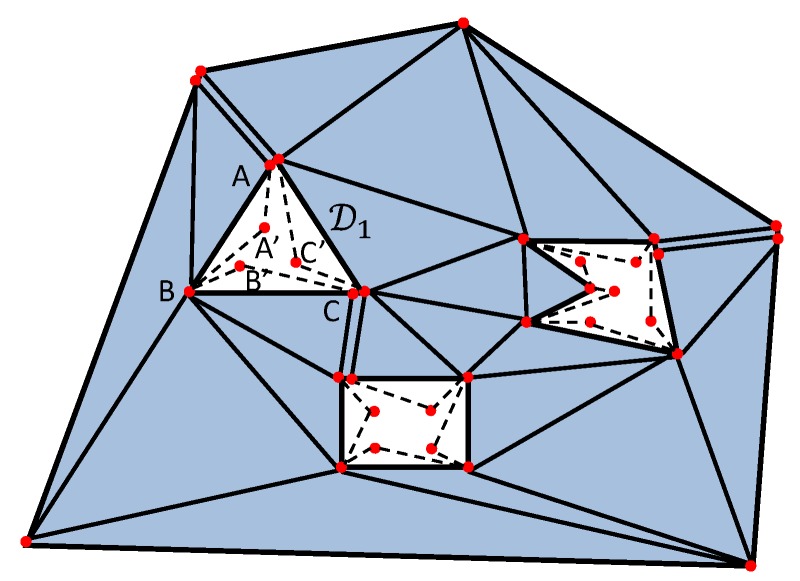
The construction of the triangulation $\hat{T}$. A, B, C are vertices of $D_{1}$ and A’, B’, C’ are vertices of $E_{1}$.

**Step A3:** Build a triangulation $\hat{T}$ by enlarging the triangulation T by adding some triangles as follows. For any *i*, add all the vertices $e_{i}^{j}$ of $E_{i}$ to T. So $n_{1}+n_{2}+\cdots +n_{k}$ vertices have been added to the triangulation. $n_{1}+n_{2}+\cdots +n_{k}$ new triangles as added as follows. For any added vertex $e_{i}^{j}$, add the triangle $\left(d_{i}^{j}d_{i}^{j+1}e_{i}^{j}\right)$ where $d_{i}^{j},d_{i}^{j+1}$ are the corresponding vertices of $D_{i}$, see Figure 5.

**Step A4:** Paint the vertices of the triangulation $\hat{T}$ into three different colors so that any triangle of the triangulation has vertices of three different colors. The following is to prove that such three-coloring exists and give a method to build it. First, build the so-called dual graph of the triangulation $\hat{T}$ in which the vertices correspond to the triangles of the triangulation $\hat{T}$, and two vertices are connected by an edge if and only if the corresponding triangles have a common side. It is obvious that this dual graph is a tree. Indeed, if it is not a tree, then it has a cycle, hence there exists a hole inside of this cycle in the polygon Q, which contradicts to the fact that Q has no holes. Furthermore, since this dual graph is a tree, it should have a hanging vertex (a vertex with only one edge). Let denote this vertex $V_{1}$ and take it away from the graph. The remaining graph is a tree again, hence it has a hanging vertex. Let denote this vertex $V_{2}$ and take it away from the graph. this operation is done step by step until last vertex $V_{M}$ of the dual graph is got. Now, paint all the vertices of the triangulation $\hat{T}$ into three colors as follows. First, paint three vertices of the triangle corresponding to $V_{M}$ into three different colors. Then for i=1,…,M−1, if all the vertices of all the triangles corresponding to $V_{M},\ldots ,V_{M-i+1}$ have been painted, take the triangle corresponding to $V_{M-i}$. By the above construction, this triangle has a common side with one of the triangles corresponding to $V_{M},\ldots ,V_{M-i+2},V_{M-i+1}$ and no common sides with all other of these triangles. The two vertices corresponding to the common side have already been painted (they are vertices of the triangle corresponding to some $V_{j}$ where M≥j≥M−i+1. Now paint the third vertex into the third remaining color. So, step by step all the vertices of the triangulation $\hat{T}$ have been painted.

**Step A5:** The set of vertices of the triangulation $\hat{T}$ is now divided into three non-intersecting subsets corresponding to three different colors. Among these three subsets, take the one with the minimum number of vertices. Place the drones at points with (x,y) coordinates corresponding to the vertices of this subset. If some vertex is a vertex of a cutting diagonal, which now corresponds to two vertices of the polygon Q, only one drone is placed at that vertex.

**Step A6:** Select the altitude of each drone as follows. If the corresponding vertex is not a vertex of any polygons $D_{i},E_{i}$, let $d_{l}$ be the maximum of the lengths of all the triangulation triangles sides for which this vertex is one of the two end points. Then place a drone at this vertex at the altitude

(5) $$z_{d}:=\max\left\{Z_{min},\epsilon +\frac{d_{l}}{\tan(\frac{\alpha }{2})}\right\}.$$

If the corresponding vertex is a vertex of some polygons $D_{i},E_{i}$, let ${\hat{d}}_{l}$ be the maximum of the lengths of all the triangulation triangles sides for which this vertex is one of the two end points and the distances to all the vertices of the two side quadrilaterals for which this point is a vertex. Moreover, let *a* be the maximum altitude of all the terrain points corresponding to the vertices of the two side quadrilaterals for which this point is a vertex. Furthermore, let $d_{m}$ be the maximum distance from this vertex to the vertices of the corresponding top side polygon $E_{i}$, and *b* be the maximum altitude of all the terrain points corresponding to the vertices of $E_{i}$ and the two corresponding side quadrilaterals. Also, let ${\hat{d}}_{m}:=\max\left\{{\hat{d}}_{l},d_{m}\right\}$. Then for each *i*, select one vertex that is a vertex of some polygon $D_{i}$ or $E_{i}$ and place a drone at this vertex at the altitude

(6) $$z_{d}:=\max\left\{Z_{min},b+c_{2}+\frac{{\hat{d}}_{m}}{\tan(\frac{\alpha }{2})}\right\}.$$

For all other selected vertices that are vertices of either polygon $D_{i}$ or $E_{i}$, place a drone at this vertex at the altitude

(7) $$z_{d}:=\max\left\{Z_{min},a+c_{2}+\frac{{\hat{d}}_{l}}{\tan(\frac{\alpha }{2})}\right\}.$$

**Remark** **1.** *It should be pointed out that in the proposed algorithm* **A1** *–* **A6** *, this approach always considers triangulations on the ground plane z=0. So, all vertices of triangulations are projections of the points on the actual uneven terrain, and the lengths of triangulations sides and quadrilaterals diagonals are also taken on the plane, not on the actual terrain.*

**Remark** **2.** *In the case when two selected vertices on the triangulation correspond to the same point of the actual terrain, which can happen when either there are two vertices from one which is the end point of a cutting diagonal in* **A1** *or a vertex of $D_{i}$ coincides with a vertex of $E_{i}$, place just one drone at the point corresponding these two vertices.*

**Remark** **3.** *Step* **A4** *of the algorithm is based on the 3-coloring method of [34].*

For the main result, the following assumptions are needed.

**Assumption** **4.** *The drone altitudes $z_{d}$ defined by (5)–(7) satisfy $z_{d}\leq Z_{max}$.*

**Assumption** **5.** *The length d of any side of any triangle of the triangulation T satisfies $d\geq c_{1}$.*

The following notation is also needed. For any number x≥0, ⌊x⌋ denotes the integer part of *x*, i.e., the maximal integer *i* such that i≤x.

Now it is the position to state the main result of the paper.

**Theorem** **1.** *A number N of drones are deployed by the algorithm* **A1** *–* **A6** *. Suppose that Assumptions 1–5 hold. This deployment is covering and*

(8) $$N\leq \lfloor \frac{n+2n_{1}+\cdots +2n_{k}+2k}{3}\rfloor .$$

**Proof** **of** **Theorem** **1.** It is obvious that the number of vertices in the constructed triangulation $\hat{T}$ is $n+2n_{1}+\cdots +2n_{k}+2k$. Since all these vertices are painted into three colors and take a color with the minimum number of vertices, the number of these vertices *N* satisfies (8). Now prove that this deployment of drones is covering. Indeed, by the construction, any triangle of $\hat{T}$ has a drone deployed at one of its three vertices. Furthermore, any point outside of the very uneven areas belongs to one of the triangles of the triangulation T, and the drone altitudes (5)–(7) and Assumption 1 guarantee that this point is visible from the drone that is located at one of the three vertices of this triangle. Moreover, it obviously follows from the construction that for any side face of any very uneven area, there is a drone deployed at one of its four vertices. The drone altitude selection rules (6) and (7) guarantee that any point of the side face is visible from a drone located at one of these four vertices. Furthermore, it is obvious that any point of the top face of any very uneven area is visible from the drone with altitude selected by (7). Moreover, for any drone location $\left(x_{d},y_{d},z_{d}\right)$, by the construction $(x_{d},y_{d})\in D$, it follows from (5)–(7) that $z_{d}\geq Z_{min}$, and Assumption 4 guarantees that $z_{d}\leq Z_{max}$. Therefore, the requirements (1) hold. Finally, (5)–(7) and Assumptions 2–5 imply that the requirements (2) are satisfied. This completes the proof of Theorem 1. □

## 4. Simulation Results

**Figure 6:**
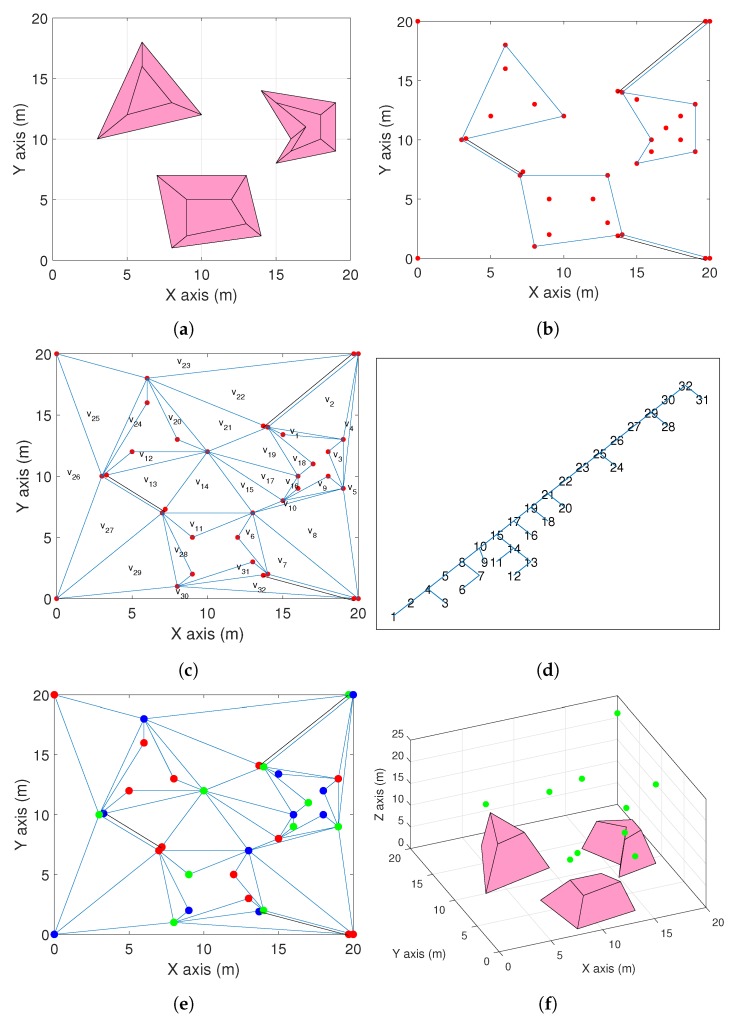
(**a**) A square area with 3 very uneven areas. (**b**) The construction of polygon Q. (**c**) The construction of triangulation $\hat{T}$. (**d**) The dual graph of $\hat{T}$ with the vertices numbered from 1 to 32. (**e**) Painting the vertices. (**f**) Deployment of aerial drones in 3D space.

This section demonstrates how the proposed approach works through a case study using MATLAB. Consider a 20 m by 20 m square area of interest shown in Figure 6a with k=3 very uneven area (a 3D view of them is available in Figure 6f). It can be seen from the top view in Figure 6a that $n_{1}=3$, $n_{2}=4$, $n_{3}=5$. The parameters are set as $c_{1}=1$ m, $c_{2}=0.5$ m, ϵ=0.2 m, $Z_{min}=4$ m, and $\alpha =\frac{\pi }{2}$. Firstly, k=3 non-intersecting diagonals are added to construct the polygon Q, see Figure 6b. It is worth pointing out that there is more than one option to insert this kind of diagonals and only one option is demonstrated here. Clearly, according to the proposed constructed, these diagonals build a Q with no holes. Then, following Step **A2** and **A3**, the triangulation $\hat{T}$ is constructed as shown in Figure 6c. This triangulation has 34 ($n+2n_{1}+2n_{2}+2n_{3}+2k$) vertices and 32 ($n+2n_{1}+2n_{2}+2n_{3}+2k-2$) triangles. By selecting a starting triangle, all the triangles are numbered in Figure 6c. Then, the dual graph of the triangulation $\hat{T}$ is constructed as shown in Figure 6d. The dual graph has 32 vertices corresponding to the 32 triangles of the triangulation $\hat{T}$. Following Step A4, all the vertices of the dual graph are numbered from 1 to 32 and they are painted in three colors (red, green, and blue). The numbers of vertices with these colors are 13, 10, and 11, respectively, see Figure 6e. Hence, the subset of vertices with the color of green is selected. Obviously,

$$N=10\leq \lfloor \frac{34}{3}\rfloor =11.$$

This also shows that the estimate of Theorem 1 is quite close. Finally, the altitudes of the drones are computed by (5)–(7), and the deployment of the aerial drones is shown in Figure 6f.

## 5. Conclusions and Future Research

A novel aerial drone deployment problem was introduced. In this problem, drones are to be placed over a very uneven terrain with the aim to cover every point of a given terrain area. A computationally simple algorithm was developed to give an upper estimate of the minimal number of drones necessary and calculate their locations. This problem may be viewed as a drone version of the 3D Art Gallery Problem. Simulation results demonstrated the effectiveness of the algorithm. There several possible directions in which this research may be extended. One important question is how to approximate a real uneven terrain by the terrain model proposed in this paper. Another practically important direction is to extend the developed Drone Art Gallery Theorem to the case when the drone placement region does not coincide with the ground surveillance region. Such situations happen when there are some ”no-fly zones” over which aerial drones are not allowed to be deployed for safety reasons. Another direction of future research is to apply the proposed method to surveillance and monitoring problems in which aerial drones are not steady but flying over an uneven terrain region [27]. This would naturally lead to 3D version of the so-called sweep coverage problem [40] for groups of drones and very uneven terrains.
